# Full range leadership style and its effect on effectiveness, employee satisfaction, and extra effort: an empirical study

**DOI:** 10.3389/fpsyg.2024.1380935

**Published:** 2024-07-25

**Authors:** Fernando Garzón-Lasso, Jorge Serrano-Malebrán, Sandra Arenas-Arango, Carlos Molina

**Affiliations:** ^1^School of Management, EAFIT University, Medellín, Colombia; ^2^Departamento de Administración, Universidad Católica del Norte, Antofagasta, Chile

**Keywords:** leadership, effort, efficiency, satisfaction, PLS-SEM

## Abstract

The purpose of this study is to determine if there is a positive relationship between full-range leadership and employees’ effort, efficiency, and satisfaction. A questionnaire was administered to 577 executives from Colombian companies, and the data was analyzed using a partial least squares structural equation modeling (PLS-SEM) approach. The results show that both transformational and transactional leadership have a direct and significant impact on extra effort, effectiveness, and satisfaction, with transformational leadership having the greatest impact on these factors. Conversely, passive-avoidant leadership has negative effects on these three constructs. This study validates the effectiveness of the MLQ 5X in a South American country, a geographical region where such studies are in their early stages. Finally, the whole range of leadership styles—transformational, transactional, and passive-avoidant—is looked at. These styles are seen as second-order constructs that challenge latent multidimensional models as they emerge.

## 1 Introduction

One of the most representative and influential models in modern organizations is the full-range leadership model that Bass and Avolio (2000a,b) proposed. This model encompasses elements of transformational, transactional, and passive-avoidant leadership styles (Leigh et al., 2021). This nine-dimensional model has formed the foundational basis for research on new leadership models (Antonakis et al., 2003) and has enabled a better understanding of the effects of leadership styles on employees’ extra effort, effectiveness, and satisfaction in organizational leadership contexts. Its core premise revolves around the idea that by inspiring employees to connect their personal purpose and life meaning with that of the organization, they achieve levels of fulfillment that exceed their own expectations. By performing their tasks with an interest beyond a mere transaction, employees put in additional efforts, consequently achieving greater efficiency and satisfaction in their results (Bass and Avolio, 2000a,b).

The design of this proposed research aims to test the full range of leadership theory and its relations with leadership effectiveness, extra effort, and satisfaction within a Latin American context. By attempting to falsify the Bass and Avolio (2000a,b) model, this study seeks to enhance the verisimilitude of the theory, aligning with Popper’s (2010) assertion that the ultimate goal of science is achieving greater truth likeness. This methodological approach, particularly in Colombia, emphasizes refutation over confirmation, reducing bias and error while strengthening the theoretical framework if it withstands falsification. This approach not only adds to global knowledge but also advances local understanding, underscoring the importance of falsificationism in scientific progress. According to Popper (2010), actively seeking to disprove a theory is a critical and necessary practice for the advancement of scientific knowledge.

This study provides several insights that demonstrate its relevance. Firstly, according to a literature review from 2019 to 2023 in Web of Science and Scopus, this is one of the few studies that utilizes structural equations to analyze the data. All of the results are significant and align with findings from other studies when examining the three leadership models and the three outcome variables. Finally, the study challenges latent multidimensional models by utilizing three second-order constructs (transformational, transactional, and passive-avoidant) as emerging multidimensional models to describe three distinct leadership styles (Batista-Foguet et al., 2021). While several studies have focused on the relationship between full-range leadership and employees’ effort, efficiency, and satisfaction, we know that few studies have actually shown a positive and significant relationship between these variables. This, in addition to validating the postulates of Bass and Avolio (2000a,b), facilitates a more comprehensive understanding of leadership, particularly in Latin America, where these studies are still in their infancy. This presents an opportunity to understand leaders’ management styles and their interactions with diverse cultures, which differ from those in North America, Europe, and Asia. However, their economic exchanges with developed countries are highly interconnected. Additionally, it demonstrates the adaptability and reliability of the theory in different contexts. Similarly, it enables organizations to enhance their competitiveness in increasingly complex and challenging environments. Finally, in addition to validating the theory, it allows for consideration of its evolution, where its adaptation as a full-range model to green or sustainable organizational contexts, increasingly demanded by society, is still in its early stages. Antonakis et al. (2003) recommend confirming the full range of the leadership model and the different effects in a variety of contexts.

The purpose of this study is to thoroughly investigate if there is a positive relationship between full-range leadership and employees’ effort, efficiency, and satisfaction in a Latin American country, as suggested by the theoretical and normative models about passive-avoidant, transactional, and transformational leadership. To achieve this objective, the study primarily addresses the following question: Is there a positive relationship between full-range leadership and employees’ effort, efficiency, and satisfaction among managers who work in organizations located in Colombia?

Colombian work culture features a unique blend of hierarchical structure, interpersonal relationships, and collectivist principles that significantly influence leadership styles and organizational dynamics. The Hofstede model, widely referenced since its introduction in 1980, categorizes culture along six dimensions—power distance, individualism versus collectivism, masculinity versus femininity, uncertainty avoidance, long-term orientation, and indulgence (Hofstede, 2011). These dimensions provide insights into the cultural forces shaping business practices in Colombia, valuable for both local and multinational firms (Poturak et al., 2020; Chun et al., 2021; The Culture Factor Group, 2023).

In Colombia, a high power distance is evident with strong respect for authority and hierarchical structures, where subordinates are unlikely to challenge leaders. This is complemented by a pronounced collectivist ethos emphasizing teamwork and loyalty over individual achievements. Despite a competitive drive, Colombians value collective affiliations and group achievements over personal gain, often prioritizing work commitments over leisure. There is also a preference for clear regulations and stability, requiring leaders to provide explicit guidance, although plans may not always be meticulously executed. Businesses generally exhibit a short-term orientation, focusing on immediate results and upholding traditional values, while a high indulgence score indicates a cultural emphasis on enjoying life, leading to work environments that prioritize positivity and social cohesion (Varela et al., 2010; Cabeza et al., 2013; Tarapuez-Chamorro et al., 2021; The Culture Factor Group, 2023).

This research aims to help contemporary organizations understand how and why this model can serve as a guiding light, illuminating the actions and behaviors of individuals who exercise leadership roles within organizations effectively, with integrity, and in a transformative manner. This, in turn, contributes positively to the sustainable growth of both the organization and the world. Researchers and employees use the Multifactor Leadership Questionnaire (MLQ 5X) to find out about leadership in a variety of settings (Bajcar and Babiak, 2022; Wang et al., 2023). The MLQ 5X includes scales for effectiveness, satisfaction, extra effort, and idealized influence, as well as constructs for *laissez-faire*, passive management by exception, active management by exception, contingent reward, individualized consideration, intellectual stimulation, inspirational motivation, and idealized influence. First, it involves surveying over 500 individuals in managerial positions within organizations located in Colombia, making it one of the few studies conducted in a Latin American developing country.

This study first provides a theoretical framework on the theory of transformational leadership and full-range leadership, connecting the three leadership styles with the outcome variables (employee effort, efficiency, and satisfaction). Subsequently, the fieldwork is conducted, and the data is analyzed using structural equations. Finally, the results are discussed, and conclusions are presented.

## 2 Literature review

### 2.1 Full-range leadership

In response to what Burns (1978) termed a leadership crisis associated with intellectual mediocrity, he published “Leadership,” a book now considered the genesis of transformational leadership theory. It was approached from a sociological and humanistic psychology perspective, clearly differentiating between leadership and tyranny. Burns (1978) characterized leadership as a social process that is part of the dynamics of conflict and power, distinct from raw power, and influenced by leaders and followers who share a common purpose. Within leadership, he argued for the existence of two basic types: transactional leadership and transformational leadership (Burns, 1978; Bass and Avolio, 2000a,b). Burns’ proposal is classified as part of the neo-charismatic theories, which also include servant leadership (Greenleaf, 1977; McGhee, 2023) and charismatic leadership (House, 1976). Over the past decades, his proposal has become the most researched theory by academics and has been considered the most appropriate leadership style for contemporary organizations. It has also served as a basis for studies on leadership behavior and its impact on organizations (Lerutla and Steyn, 2022).

Following the conduct of multiple empirical studies, Bass’s (1985) proposal evolved into the Full Range Leadership Model formulated by Bass and Avolio (1994), which currently proposes the existence of three leadership styles: (a) passive-avoidant leadership, characterized by *laissez-faire* behaviors and passive management by exception; (b) transactional leadership, with the factors of active management by exception and contingent reward; and (c) transformational leadership, comprised of behaviors like individualized consideration, intellectual stimulation, inspirational motivation, and idealized influence (see Supplementary Figure S1).

Burns (1978) argued that transactional leadership is more commonly used in leader-follower relationships, as it focuses on the exchange between parties seeking individual benefits, such as task completion, in exchange for compensation. Based on Maslow and Kholbergh, he defined transformational leadership as that which seeks to satisfy higher-order needs and results in mutual stimulation and elevation, turning followers into leaders and possibly transforming leaders into moral agents (Bass, 1985). Bass (1985) supported the argument for the existence of two fundamental leadership styles—transactional and transformational—by adding to Burns’ (1978) proposal. However, in contrast to Burns (1978), these styles are seen as complementary to each other (Changar and Atan, 2021), contributing in different ways to the fulfillment and surpassing of performance expectations within organizations. Transactional leadership provides clarity in goals and seeks to meet resource needs for their accomplishment, involving behaviors associated with basic structural or task-oriented approaches. On the other hand, transformational leadership offers individualized consideration, fostering the development of team members by satisfying higher-order needs. It includes intellectual stimulation that democratizes the search for alternative solutions to challenges and inspiration based on a common purpose and the charisma of the leader.

### 2.2 Relationship of transformational leadership with effort, efficiency, and employee satisfaction

Transformational Leadership is deemed to be the most appropriate style of leadership in contemporary organizations and the most ideal form of organizational leadership (Berber et al., 2019). In this approach, the individual tends to take actions to increase awareness of what is right, good, and important, aiming to enhance the motivational maturity of followers and encourage them to go beyond their self-interests in pursuit of the group’s, organization’s, and society’s well-being. Transformational leaders strive to elevate the achievement, motivation, and self-development of employees, generating strong identification and trust among team members. Therefore, they inspire them to reach their potential in the pursuit of personal self-actualization with high ethical and moral standards (Avolio and Bass, 2004; Poturak et al., 2020; Garzón-Lasso et al., 2021) and increase productivity as a way to generate competitive advantage (Adunola et al., 2023). This leadership comprises the styles of individualized consideration (IC), intellectual stimulation (IS), inspirational motivation (IM), idealized influence through behavior (IIB), and idealized influence through attributes (IIA), the descriptions of which are presented below (Bass and Avolio, 1994).

#### 2.2.1 Individualized consideration

Individualized consideration involves attending to team members’ needs and striving to develop their full potential. Leaders focus on each individual’s need for achievement and growth, acting as coaches or mentors. They create new learning opportunities in a supportive environment, recognize individual differences in needs and desires, and assist followers in reaching higher levels of potential (Bass and Avolio, 1994; Yukl and Uppal, 2017).

#### 2.2.2 Intellectual stimulation

This style promotes innovative thinking within the team by encouraging continuous questioning and idea generation. Leaders foster creativity and innovation by challenging assumptions, redefining problems, and re-evaluating past situations. They treat errors as learning opportunities, focusing on identifying root causes to find future solutions. Moreover, it encourages followers to seek creative ideas and solutions using collective methodologies, design thinking, and analysis. Leaders understand the imperfection of human actions and emphasize continuous improvement, leveraging the collective intelligence of the team to establish challenges and alternatives (Bass and Avolio, 1994; García et al., 2011; Yukl and Uppal, 2017).

#### 2.2.3 Inspirational motivation

Inspirational motivation is when leaders serve as an inspiration and motivational figure for their team. They articulate a shared vision of goals and what is right and important, promoting emotional intelligence within the team to help them face challenges without losing motivation. These leaders behave in ways that give meaning and challenge to their followers’ work, reviving individual and team spirit with enthusiasm and optimism. They encourage followers to envision a better future for the organization and themselves (Bass and Avolio, 1994; García et al., 2011; Yukl and Uppal, 2017).

#### 2.2.4 Idealized influence through attributes

Idealized influence through attributes focuses on conveying trust to followers and seeking their identification with the leader. Such leaders inspire pride in their team members by prioritizing common goals over personal interests (Bass and Avolio, 1994; García et al., 2011; Yukl and Uppal, 2017).

#### 2.2.5 Idealized influence through behaviors

Regarding this style, leaders act with integrity and exhibit valued behaviors such as dominance, conscientiousness, self-control, moral judgment, and self-sufficiency (Bass and Avolio, 1994; García et al., 2011; Yukl and Uppal, 2017).

Transformational leadership, in addition to having a positive impact on employee effort, efficiency, and satisfaction, is the style most associated with group performance (Avolio and Bass, 2004), serving as an inspiration that leads workers to extraordinary performance levels (Teoh et al., 2022). These connections are in line with the findings of a study by Alhuzaim et al. (2022), which examined the effects of school directors’ transformational leadership styles on 463 teachers and 24 public and private school directors in Saudi Arabia. Similarly, Mgaiwa (2023) posits, in his study, that transformational leadership has a strong relationship with the job satisfaction of academics in Tanzania. Lastly, Hitch et al. (2020) also found, in a study conducted with 93 occupational therapists in Australia, a positive relationship between transformational leadership and extra effort, effectiveness, and satisfaction. Based on all the aforementioned, it is possible to develop the following hypotheses.

Transformational leadership, in addition to having a positive impact on employee effort, efficiency, and satisfaction, is the style most associated with group performance (Avolio and Bass, 2004; Poturak et al., 2020), serving as an inspiration that leads workers to extraordinary performance levels (Teoh et al., 2022). Hitch et al. (2020) conducted a study with 93 occupational therapists in Australia and found a positive relationship between transformational leadership and extra effort, effectiveness, and satisfaction. Adunola (2023) conducted another study where 235 nurses evaluated their management nurses in a hospital in Nigeria, which shows positive and significant relationships among transformational leadership and outcomes. These connections are also in line with the findings of a study by Alhuzaim et al. (2022), which examined the effects of school directors’ transformational leadership styles on 463 teachers and 24 public and private school directors in Saudi Arabia. Similarly, Mgaiwa (2023) asserts in his study that transformational leadership has a strong relationship with academic job satisfaction in Tanzania. Drawing from the aforementioned information, we can formulate the following hypotheses:

*H1*: Transformational leadership styles have a direct and positive relationship with extra effort.

*H2*: Transformational leadership styles have a direct and positive relationship with effectiveness.

*H3*: Transformational leadership styles have a direct and positive relationship with employee satisfaction.

### 2.3 Relationship of transactional leadership with effort, efficiency and employee satisfaction

In transactional leadership (TL), leaders exhibit behaviors associated with two transaction styles: constructive (rewarding achievements) and corrective (controlling deviations and errors). This leadership style defines expectations, promotes performance to achieve agreed-upon goals, and monitors deviations and errors (Avolio and Bass, 2004; Garzón-Lasso et al., 2021). The behaviors of Active Management by Exception focus on supervising deviations and errors, continuously monitoring the performance of their team, concentrating their attention on mistakes made, and seeking to correct them. These leaders specify compliance standards, as well as what constitutes ineffective performance, and may punish followers for not meeting these standards. This leadership style involves close monitoring of deviations, errors, and failures and the immediate implementation of corrective measures. As for contingent reward (CR) behaviors, leaders reward achievements, clarify expectations, and offer recognition when goals are met. They also tend to monitor performance and motivate their followers through the exchange of rewards. This type of behavior usually provides the necessary support for meeting goals, depending on the needs of their team. When assigning a task, they might be inclined to clearly establish objectives and responsibilities.

Research has revealed a contradictory relationship between transactional leadership style (TLS) and job performance. Several studies have found that transactional leaders use rewards to get employees to work harder (Humphreys, 2001; Voon et al., 2011; Mahdinezhad et al., 2013; Shah and Hamid, 2015; Aymerich et al., 2021; Changar and Atan, 2021; Wegner, 2024), and some have found that the TLS style has a big and positive effect on getting employees to work harder (Sundi, 2013; Changar and Atan, 2021). However, other studies have found a negative relationship between TLS and employee performance. In further studies, TLS has been found to have a positive and significant relationship with employee motivation (Chaudhry and Javed, 2012; Fjendbo, 2021). Additional research has indicated that TLS promotes stability and maintenance of the status quo, as leaders set goals for their followers and reward them for meeting expectations (Xenikou, 2017; Lerutla and Steyn, 2022).

According to Avolio and Bass (2004), transactional leadership is associated with favorable outcomes in these three variables (effort, efficiency, and employee satisfaction). This is in line with the findings of the study by Alhuzaim et al. (2022), which showed that this leadership style has a positive effect on effort, effectiveness, and employee satisfaction. Another study in Tanzania showed a positive, albeit weak, relationship between academic job satisfaction and transactional leadership, which could be due to its complementarity with transformational leadership (Mgaiwa, 2023). Finally, a study conducted in Australia with 93 occupational therapists also found significant positive relationships between transactional leadership and extra effort, effectiveness, and satisfaction (Hitch et al., 2020). Based on all the aforementioned, the following hypotheses can be developed:

*H4*: Transactional leadership styles have a direct and positive relationship with extra effort.

*H5*: Transactional leadership styles have a direct and positive relationship with effectiveness.

*H6*: Transactional leadership styles have a direct and positive relationship with employee satisfaction.

### 2.4 Relationship of passive-avoidant leadership with effort, efficiency and employee satisfaction

Leaders with high frequencies of passive or avoidant behaviors tend to fail in identifying and clarifying potential problems and avoid getting involved and monitoring outcomes (Antonakis et al., 2003). These leaders often do not respond to complex situations that may arise, and most of the time, this style has a negative effect on results, being judged as ineffective (Lerutla and Steyn, 2022). Similarly, they do not systematically respond to situations and problems. They are also characterized by their avoidance of specifying agreements, clarifying expectations, and providing objectives and standards (Avolio and Bass, 2004; Garzón-Lasso et al., 2021). Regarding the *laissez-faire* (LF) dimension, leaders who adopt this style avoid involvement, engagement, and assuming responsibilities. They may fail to provide sufficient information to achieve their objectives or not offer appropriate feedback to their team. This leadership style could easily be defined as “non-leadership.” These permissive leaders refuse to assume leadership responsibilities, do not offer sufficient information to their followers, do not set goals to be achieved, do not provide feedback, and do not recognize or work towards the satisfaction of their followers.

On the other hand, behaviors associated with passive management by exception (PME) range from being passive in the face of problems, intervening only when they become serious, to firefighting within their team or organization, waiting for a problem to arise before taking corrective measures. In this style, corrective action is often punitive. According to Berber et al. (2019), passive-avoidant leadership has a negative impact on outcomes in terms of extra effort, effectiveness, and satisfaction (Alhuzaim et al., 2022). Their research demonstrated a negative relationship between passive-avoidant leadership and extra effort, effectiveness, and satisfaction. Based on all the aforementioned, it is possible to develop the following hypotheses:

*H7*: Passive-avoidant leadership styles have a direct and negative relationship with extra effort.

*H8*: Passive-avoidant leadership styles have a direct and negative relationship with effectiveness.

*H9*: Passive-avoidant leadership styles have a direct and negative relationship with employee satisfaction.

Supplementary Figure S2 depicts the proposed model for this research.

## 3 Methodology

### 3.1 Sampling

The data were collected through a survey conducted between June and November 2022 in companies in Colombia. The survey targeted 589 participants with managerial roles in their organizations. Convenience sampling was used; the respondents were participants in leadership education programs. To prevent common method variance bias, the study design included participants from different leadership programs at different times. Additionally, the surveys were completed before the participants began their training process in a classroom setting, and professional interviewers conducted the surveys. To eliminate potential ambiguities in the instrument, a pilot test was conducted with the first leadership course, which included 40 participants. Face-to-face surveys were conducted in 20 groups of leadership education programs. The exclusion of invalid surveys resulted in a final sample of 577 respondents, of which 50.1% were male and 49.9% were female. The average age was 37 years. Most participants had undergraduate education, and the economic sectors with the highest number of participants were services, commerce, and industry (Supplementary Table S1).

### 3.2 Measurement scales

The MLQ5X 36-item questionnaire was utilized to measure transformational, transactional, and *laissez-faire* leadership styles (Avolio and Bass, 2004). Specifically, the Spanish version of the Multifactor Leadership Questionnaire, translated and licensed by MindGarden Inc., was used. It comprises nine dimensions: (1) idealized influence attributed (IIA), representing the attribution of charisma; (2) idealized influence behavior (IIB), reflecting the behavioral aspect of charisma; (3) inspirational motivation (IM), relating to the leader’s thought-provoking and motivating behavior; (4) intellectual stimulation (IS), denoting stimulating followers towards unconventional and creative thinking; (5) individualized consideration (IC), demonstrating genuine interest in each follower’s well-being and attending to their individual needs; (6) contingent reward (CR), representing fair and constructive management processes for rewarding good performance, both financially and psychologically; (7) active management-by-exception (MBEA), reflecting active monitoring of followers’ work and taking corrective actions when necessary; (8) passive management-by-exception (MBEP), describing leader’s intervening behaviors upon the occurrence of problems; and (9) *laissez-faire* (LF), expressing the absence of leadership or lack of involvement in leading (Avolio and Bass, 2004).

### 3.3 Statistical tools

A structural equation modeling (SEM) approach, specifically partial least squares (PLS), is proposed to test the reliability, validity, and hypotheses. PLS-SEM allows researchers to assess both causal relationships between indicators and items and causal relationships between latent constructs (Gudergan et al., 2008). To evaluate the measurement models and the structural model, procedures suggested in prior literature were used (Fornell and Larcker, 1981; Wright et al., 2012; Henseler et al., 2016). The data were analyzed using the SmartPLS 4 software (Ringle et al., 2022).

## 4 Results

Wright’s et al. (2012) proposed steps for estimating a component-based model as the basis for the process used to obtain the results. The process involves: (1) running the first-order model; (2) assessing reliability; (3) evaluating convergent validity; (4) assessing discriminant validity; (5) creating a new data file with the scores of the latent variables; (6) constructing the second-order factor with the latent variables as indicators; (7) running the structural model; and finally, (8) evaluating the results of the structural model.

### 4.1 Measurement model evaluation

To analyze the instrument, criteria for reliability, convergent validity, and discriminant validity were evaluated. There are three indicators in Supplementary Table S2: the Cronbach’s alpha coefficient (CA), the composite reliability (CR), and the average variance extracted (AVE) for each construct. The results of Cronbach’s alpha (CA) and composite reliability (CR) ensure the reliability of the scales. The results of Cronbach’s alpha are in a range between 0.847 and 0.936, above the recommended value of 0.7 for scale robustness, and the composite reliability of the proposed model varies between 0.907 and 0.959, surpassing the recommended value of 0.7 (Henseler et al., 2016). This indicates that the constructs have a high level of internal consistency. To assess convergent validity, the loadings of each item and the AVE were examined. The loadings of each item were greater than 0.7 (Fornell and Larcker, 1981). The average variance extracted ranges between 0.718 and 0.886, higher than the accepted level of 0.5 (Chin, 1998). These results suggest adequate convergent validity for all the latent constructs.

To assess discriminant validity, the Fornell–Larcker criterion (Fornell and Larcker, 1981) and HTMT ratio (Voorhees et al., 2016). Firstly, Fornell and Larcker (1981) suggest that discriminant validity can be evaluated by examining whether the square root of the AVE is greater than the correlations with other constructs. As shown in Supplementary Table S3, all values on the diagonal exceed the correlations between constructs. Additionally, Supplementary Table S4 shows the HTMT criterion, which is a ratio of within-construct correlations to between-construct correlations. The HTMT values are below the required value of 0.9 (Henseler et al., 2016). In conclusion, the results indicate adequate discriminant validity.

### 4.2 Second-order constructs evaluation

We looked at the loadings, average variance extracted, composite reliability, and Cronbach’s alpha of the latent scores of the first-order constructs to figure out what the higher-level PLS constructs were like. The statistical analysis presented in Supplementary Table S5 assesses the second-order constructs of transformational leadership, transactional leadership, and passive-avoidant leadership.

### 4.3 Structural model evaluation

The steps suggested by Wright et al. (2012) were used to test the structural model with the second-order variable. To do this, aggregated scores were used to model the second-order construct. The goodness of fit should be assessed at the beginning of the model evaluation before examining the structural model (Henseler et al., 2016). To assess goodness of fit, use SmartPLS4 software (Ringle et al., 2022), which provides the standardized root mean square residual (SRMR) as the appropriate measure for model fit. For the proposed model, the SRMR value is 0.047, which indicates a good fit of the model (Prasarnphanich and Wagner, 2009). The proposed structural model is evaluated through path loadings and R-squared (*R*^2^) values. As suggested by Streukens and Leroi-Werelds (2016), PLS bootstrapping with 10,000 samples was used, and path loadings and *p*-values were found for the relationships in the hypotheses. The results are shown in Supplementary Figure S3. Supplementary Table S6 shows the hypothesis’s findings, with appropriate path coefficients and p-values supporting all suggested direct relationships.

The results show that all hypotheses are supported by their sign and level of significance. Supplementary Table S6 indicates that transformational leadership has the strongest effects, followed by transactional leadership. As expected, Passive-Avoidant leadership has a negative effect on job satisfaction, extra effort, and worker effectiveness. The *R*^2^ values depicted in Supplementary Figure S2 suggests that the model explains 57.6% of extra effort, 43.6% of effectiveness, and 49.4% of satisfaction.

## 5 Discussion

The main objective of this study is to determine if there is a positive relationship between full-range leadership and employees’ effort, efficiency, and satisfaction which was reached through the methodology used. Based on hypothesis postulated, the following analyses can be drawn: first, the results obtained also demonstrate that both transformational and transactional leadership have a direct, significant, and positive relationship with extra effort, effectiveness, and satisfaction. This confirms the proposal made by Bass (1985) and Bass and Avolio (2000a,b) regarding the complementarity between both leadership constructs to achieve positive effects in organizations (Changar and Atan, 2021). According to Avolio and Bass (2004), the establishment of feedback or recognition mechanisms and the clear identification of roles and goals in transactional leadership serve as the foundation that enables their fulfillment and, as a result, have a positive, albeit moderate, relationship with effectiveness (0.18), extra effort (0.14), and satisfaction (0.17), with their impacts being quite similar.

Second, when examining the relationships between each dependent construct and transformational, transactional and passive-avoidant leadership, Transformational leadership has the greatest impact on extra effort, effectiveness, and satisfaction, while transactional leadership has the least. These results are consistent with the study by Martínez-Moreno et al. (2021), who found that transformational leadership has a greater impact on employees’ extra effort, effectiveness, and satisfaction, followed by transactional leadership. It is important to continue to enhance transformational leadership, whose leaders are considered examples of ethics and morality, to have more motivated and committed employees (Londono-Proano, 2022). In other words, it is crucial for leaders, depending on the situation, to complement transformational leadership with transactional leadership, serving as a source of inspiration for their workers and, additionally, establishing goals related to reward or punishment systems (Changar and Atan, 2021).

Third, the results also support the idea that passive-avoidant behaviors have negative effects on leadership outcomes in Colombian companies, as shown by the fact that they make leaders less effective (−0.247), less satisfied (−0.141), and less hardworking (−0.120) (Berber et al., 2019). These results are consistent with their avoidance of involvement, assuming responsibilities, and giving objectives and standards (Garzón-Lasso et al., 2021).

Fourth, the results also identify that transformational leadership has a greater impact on extra effort (0.594), satisfaction (0.458), and effectiveness (0.421) than transactional leadership. The ability of this leadership style to connect with followers’ higher-order needs explains this, which is consistent with earlier studies (Berber et al., 2019; Alhuzaim et al., 2022). Transformational leaders also have the capacity to enhance employees’ satisfaction with their job and the aspects surrounding it (Abolnasser et al., 2023). The individualized consideration dimension, which values team members’ ideas in identifying problems and coming up with solutions and describes it as intellectual stimulation, the sense of tasks and common purpose of teams described as inspirational motivation, and integrity and trust-building in leadership through idealized influence, leading followers to achieve extraordinary performance, facilitate this (Teoh et al., 2022).

In addition to justifying the hypotheses, the results of this study validate the use of the MLQ 5X in its Spanish version to measure the nine dimensions of the full range proposed by Bass and Avolio (2000a,b) in Latin America. This contributes to advancing the understanding of the model in different contexts (Antonakis et al., 2003; Becerra-Astudillo et al., 2022) and addresses the need to comprehend the effects of the three leadership styles.

In addition to the above, this research confirms the existence of second-order constructs, supporting what Batista-Foguet et al. (2021) have postulated. Along with analyzing the validity of the MLQ 5X, it examines causal relationships. According to Bass and Avolio (2000a,b), the dimensions of *laissez-faire* and passive management by exception make up a second-order construct known as passive-avoidant leadership. Meanwhile, active management by exception and contingent reward make up the construct called transactional leadership. Finally, the dimensions of individualized consideration, intellectual stimulation, inspirational motivation, idealized influence behavior, and idealized influence attributed form the second-order construct called transformational leadership.

Although the results align with the postulates of Bass and Avolio (2000a,b), it would be prudent to conduct a replication of this study in a different Latin American country to confirm whether the data supports the validity of the theory. Additionally, some control variables, such as gender, could be incorporated in the future to verify if the results remain consistent with the theoretical postulates.

## 6 Theoretical and practical implications

### 6.1 Theoretical implications

This study presents several theoretical implications. First, it backs up what Bass and Avolio (2000a,b) found about the full-range leadership model. It shows that transformational, transactional, and passive-avoidant leadership all lead to more effort, effectiveness, and satisfaction, which is something that many other studies have not seen. Additionally, it demonstrates that transformational leadership has the greatest impact on its outcomes, thus underscoring its importance in a developing country. Additionally, it validates the Multifactor Leadership Questionnaire (MLQ 5X) in a unique context, given its predominant application in developed countries. It is one of the few studies between 2019 and 2023 that uses structural equations to analyze the data, according to WOS and Scopus. Finally, it challenges not only latent multidimensional models with second-order constructs, but also emerging multidimensional models.

### 6.2 Practical implications

The practical implications of this study are equally numerous. Firstly, it confirms the importance of promoting transformational leadership in organizations due to its greater impact on outcomes. Furthermore, the positive impact of this style on organizational productivity underscores the need for companies to align their organizational leadership in this direction to maintain their competitiveness and sustainability in the long run. Organizations should also promote workshops that reinforce transformational leadership, acknowledging that leaders are not just born but also created. Finally, it emphasizes the need for organizations to measure their managers’ leadership styles, ideally through the MLQ 5X, due to its high reliability. The findings shed light on how to improve their leadership abilities.

## 7 Conclusion

This study contributes to providing global validity to the MLQ because, while most studies have been conducted in developed countries, research in developing countries has emerged over time, with South America and Colombia being an area that lacked research in this field. Unfortunately, long-term research projects that provide a comprehensive understanding of leadership in Colombia are still lacking. “Global Studies” (1999) is perhaps the only significant source of information on Colombian culture and leadership. The research conducted on this variable is primarily a compilation of the authors’ experiences and beliefs rather than the product of quantitative research. Therefore, verifying that the three leadership styles, as perceived by Colombian executives, significantly impact the second-order constructs represents a valuable contribution to knowledge. Furthermore, it is one of the few studies, according to the literature reviewed between 2019 and 2023 in Web of Science and Scopus, that analyzes the data using structural equations.

The study is important and useful for understanding what leadership is. It gives solid evidence of the three types of leadership suggested by the full-range model: transformational, transactional, and passive-avoidant. These styles are analyzed as second-order constructs in emerging multidimensional models to describe three different leadership styles, challenging latent multidimensional models. The results conclude that there is a direct and positive correlation between transformational and transactional styles, extra effort, effectiveness, and job satisfaction. The study also suggests using adaptive leadership, in which leaders can use behaviors and parts of both transactional and transformational leadership depending on the situation, to make organizations more effective, especially in business environments that are dynamic and always changing.

On the other hand, while there is a positive correlation between transactional leadership and the variables of extra effort, effectiveness, and satisfaction, its impact is measured compared to transformational leadership. Therefore, the adoption of transactional leadership behaviors in leadership roles may be less effective in increasing employee satisfaction and motivation unless complemented with the adoption of transformational leadership behaviors. As for passive-avoidant leadership, its ineffectiveness with the same variables results in negative effects on employee performance and attitude. Because of these results, Latin American organizations will be able to figure out what behaviors or skills managers should look for and work on. They will focus on active behaviors that are linked to transactional styles, like setting clear goals, and transformational styles, like empathy, democracy, inspiration, coherence, transparency, and credibility can lead to more motivated and satisfied employees. However, it’s important to keep in mind that context is crucial in defining the best leadership style.

To sum up, this study effectively connects theoretical concepts to practical applications, offering a robust framework that enhances our understanding of leadership effectiveness in diverse organizational settings.

## 8 Research limitations and future directions

Although this study provides valuable information, it’s important to consider its limitations when generalizing the results to different contexts than where it was conducted. It would be important to extend the research to different cultural contexts to observe how the full-range model manifests and its impact in other cultures, helping multinational companies create effective leadership strategies sensitive to their context. Future research could be conducted in other Latin American contexts to verify if the results remain significant. Additionally, given the significant gender-based samples, it could be analyzed to see if this control variable has any relevant effect on the three leadership styles Given the increased concern for being green or sustainable (Abolnasser et al., 2023; Abdou et al., 2023; Suliman et al., 2023), the same study could be adapted to green scales. Finally, it would also be helpful to learn more about how leadership works in Latin America by looking at concepts that are different from transactional, transformational, and passive-avoidant leadership. These include authentic leadership, servant leadership, adaptive leadership, ethical leadership, and more.

## Data availability statement

The raw data supporting the conclusions of this article will be made available by the authors, without undue reservation.

## Ethics statement

Ethical approval was not required for the studies involving humans because survey directed at companies who reviewed the instrument before dissemination. The studies were conducted in accordance with the local legislation and institutional requirements. The participants provided their written informed consent to participate in this study.

## Author contributions

FG-L: Conceptualization, Formal analysis, Investigation, Software, Validation, Writing – original draft, Writing – review & editing. JS-M: Formal analysis, Investigation, Methodology, Project administration, Software, Writing – original draft, Writing – review & editing. SA-A: Conceptualization, Formal analysis, Investigation, Methodology, Validation, Writing – original draft, Writing – review & editing. CM: Conceptualization, Investigation, Methodology, Project administration, Supervision, Validation, Writing – original draft, Writing – review & editing.
