# Supplementary material for: Full range leadership style and its effect on effectiveness, employee satisfaction, and extra effort: an empirical study
Source: Front Psychol. 2024 Jul 25;15:1380935. doi: 10.3389/fpsyg.2024.1380935 (PMC11307206; doi:10.3389/fpsyg.2024.1380935)
Supplement: Supplementary file 1 [file Data_Sheet_1.docx]

Supplementary Material

# Supplementary Tables

Supplementary Table 1. Demographic data

| Item | | Frequency | Percentage |
| --- | --- | --- | --- |
| Gender | Male | 289 | 50.10 |
|  | Female | 288 | 49.90 |
| Age | 19-30 years | 173 | 30.30 |
|  | 31-40 | 179 | 30.70 |
|  | 41-50 | 144 | 24.70 |
|  | 51-60 | 64 | 11.20 |
|  | 61 or more | 17 | 3.10 |
| Economic sector | Services | 196 | 33.97 |
|  | Commerce | 150 | 26.00 |
|  | Industry | 110 | 19.06 |
|  | Construction | 69 | 11.96 |
|  | Education | 40 | 6.93 |
|  | Agriculture | 12 | 2.08 |
| Education level | High school | 75 | 13.00 |
|  | Technical | 46 | 7.97 |
|  | Technological | 69 | 11.96 |
|  | Undergraduate | 214 | 37.09 |
|  | Postgraduate | 173 | 29.98 |

Supplementary Table 2. Loadings, Average Variance Extracted (AVE), Composite Reliability (CR), Cronbach's Alpha (CA).

| Construct | First-Order Construct | Item | Loading | AVE | CR | CA |
| --- | --- | --- | --- | --- | --- | --- |
| Transformational Leadership | *Idealized Attributes* | IA1 | 0.895 | 0.764 | 0.907 | 0.845 |
|  |  | IA2 | 0.888 |  |  |  |
|  |  | IA3 | 0.836 |  |  |  |
|  |  | IA4 | 0.844 |  |  |  |
|  | *Idealized Behaviors* | IB1 | 0.935 | 0.876 | 0.955 | 0.929 |
|  |  | IB2 | 0.948 |  |  |  |
|  |  | IB3 | 0.924 |  |  |  |
|  |  | IB4 | 0.879 |  |  |  |
|  | *Inspirational Motivation* | IM1 | 0.892 | 0.757 | 0.903 | 0.840 |
|  |  | IM2 | 0.874 |  |  |  |
|  |  | IM3 | 0.842 |  |  |  |
|  |  | IM4 | 0.866 |  |  |  |
|  | *Intellectual Stimulation* | IS1 | 0.923 | 0.836 | 0.939 | 0.902 |
|  |  | IS2 | 0.900 |  |  |  |
|  |  | IS3 | 0.919 |  |  |  |
|  |  | IS4 | 0.912 |  |  |  |
|  | *Individualized Consideration* | IC1 | 0.935 | 0.881 | 0.957 | 0.932 |
|  |  | IC2 | 0.955 |  |  |  |
|  |  | IC3 | 0.926 |  |  |  |
|  |  | IC4 | 0.895 |  |  |  |
| Transactional Leadership | Contingent Reward | CR1 | 0.887 | 0.811 | 0.945 | 0.922 |
|  |  | CR2 | 0.918 |  |  |  |
|  |  | CR3 | 0.931 |  |  |  |
|  |  | CR4 | 0.866 |  |  |  |
|  | Management-by-Exception (Active) | MBEA1 | 0.927 | 0.886 | 0.959 | 0.936 |
|  |  | MBEA2 | 0.950 |  |  |  |
|  |  | MBEA3 | 0.946 |  |  |  |
|  |  | MBEA4 | 0.933 |  |  |  |
| Passive-Avoidant | Management-by-Exception (Passive) | MBEP1 | 0.887 | 0.811 | 0.945 | 0.922 |
|  |  | MBEP2 | 0.918 |  |  |  |
|  |  | MBEP3 | 0.931 |  |  |  |
|  |  | MBEP4 | 0.942 |  |  |  |
|  | Laissez-Faire | LF1 | 0.866 |  |  |  |
|  |  | LF2 | 0.927 | 0.886 | 0.959 | 0.936 |
|  |  | LF3 | 0.950 |  |  |  |
|  |  | LF4 | 0.946 |  |  |  |
| Extra Effort | | EE1 | 0.796 | 0.749 | 0.842 | 0.752 |
|  |  | EE2 | 0.843 |  |  |  |
|  |  | EE3 | 0.823 |  |  |  |
| Effectiveness | | EF1 | 0.846 | 0.842 | 0.912 | 0.853 |
|  |  | EF2 | 0.859 |  |  |  |
|  |  | EF3 | 0.832 |  |  |  |
|  |  | EF4 | 0.877 |  |  |  |
| Satisfaction | | SAT1 | 0.891 | 0.762 | 0.842 | 0.732 |
|  |  | SAT2 | 0.832 |  |  |  |

Supplementary Table 3. Discriminant Validity, Fornell-Larcker Criterion

|  | IA | AB | IM | IS | IC | CR | MBEA | MBEP | LF | EE | EF | SAT |
| --- | --- | --- | --- | --- | --- | --- | --- | --- | --- | --- | --- | --- |
| IA | 0.941 |  |  |  |  |  |  |  |  |  |  |  |
| IB | 0.499 | 0.901 |  |  |  |  |  |  |  |  |  |  |
| IM | 0.523 | 0.542 | 0.936 |  |  |  |  |  |  |  |  |  |
| IS | 0.524 | 0.517 | 0.643 | 0.874 |  |  |  |  |  |  |  |  |
| IC | 0.682 | 0.650 | 0.593 | 0.554 | 0.934 |  |  |  |  |  |  |  |
| CR | 0.613 | 0.561 | 0.770 | 0.689 | 0.522 | 0.870 |  |  |  |  |  |  |
| MBEA | 0.557 | 0.516 | 0.708 | 0.625 | 0.578 | 0.743 | 0.914 |  |  |  |  |  |
| MBEP | 0.519 | 0.511 | 0.638 | 0.577 | 0.581 | 0.649 | 0.595 | 0.875 |  |  |  |  |
| LF | 0.598 | 0.562 | 0.637 | 0.652 | 0.635 | 0.752 | 0.732 | 0.590 | 0.939 |  |  |  |
| EE | 0.625 | 0.532 | 0.597 | 0.532 | 0.524 | 0.632 | 0.613 | 0.732 | 0.615 | 0.867 |  |  |
| EF | 0.554 | 0.610 | 0.633 | 0.623 | 0.629 | 0.669 | 0.587 | 0.556 | 0.631 | 0.623 | 0.932 |  |
| SAT | 0.566 | 0.595 | 0.702 | 0.612 | 0.548 | 0.678 | 0.543 | 0.632 | 0.688 | 0.577 | 0.577 | 0.842 |

Supplementary Table 4. Discriminant Validity, Heterotrait - Monotrait Ratio (HTMT) Criterion

|  | IB | IM | IS | IC | CR | MBEA | MBEP | LF | EE | EF |
| --- | --- | --- | --- | --- | --- | --- | --- | --- | --- | --- |
| IA | 0.537 |  |  |  |  |  |  |  |  |  |
| IB | 0.560 | 0.585 |  |  |  |  |  |  |  |  |
| IM | 0.588 | 0.584 | 0.723 |  |  |  |  |  |  |  |
| IS | 0.732 | 0.701 | 0.638 | 0.625 |  |  |  |  |  |  |
| IC | 0.690 | 0.636 | 0.870 | 0.816 | 0.703 |  |  |  |  |  |
| CR | 0.607 | 0.565 | 0.772 | 0.714 | 0.631 | 0.853 |  |  |  |  |
| MBEA | 0.575 | 0.570 | 0.713 | 0.678 | 0.647 | 0.762 | 0.673 |  |  |  |
| MBEP | 0.650 | 0.678 | 0.598 | 0.688 | 0.563 | 0.848 | 0.623 | 0.632 |  |  |
| LF | 0.599 | 0.536 | 0.654 | 0.459 | 0.579 | 0.848 | 0.678 | 0.663 | 0.565 |  |
| EE | 0.632 | 0.546 | 0.564 | 0.653 | 0.663 | 0.723 | 0.533 | 0.565 | 0.653 | 0.423 |
| EF | 0.689 | 0.610 | 0.489 | 0.689 | 0.678 | 0.456 | 0.654 | 0.665 | 0.463 | 0.354 |
| SAT | 0.549 | 0.598 | 0.687 | 0.654 | 0.683 | 0.532 | 0.766 | 0.653 | 0.578 | 0.468 |

Supplementary Table 5. Loadings of first-order constructs in the second-order construct.

| Second-order construct | Items | Loadng | AVE | CR | CA |
| --- | --- | --- | --- | --- | --- |
| Transformational Leadership | *Idealized Attributes* | 0.701 | 0.597 | 0.881 | 0.831 |
|  | *Idealized Behaviors* | 0.799 |  |  |  |
|  | *Inspirational Motivation* | 0.755 |  |  |  |
|  | *Intellectual Stimulation* | 0.804 |  |  |  |
|  | *Individualized Consideration* | 0.799 |  |  |  |
| Transactional Leadership | Contingent Reward | 0.869 | 0.556 | 0.783 | 0.712 |
|  | Management by Exception (Active) | 0.721 |  |  |  |
| Passive-Avoidant Leadership | Management by Exception (Passive) | 0.891 | 0.693 | 0.881 | 0.792 |
|  | Laissez-Faire | 0.769 |  |  |  |

Supplementary Table 6. Hypothesis Results

| Hypotheses | Path | Path coefficient | P-value | Result |
| --- | --- | --- | --- | --- |
| H1 | Transformational Leadership → Extra Effort | 0.594 | 0.000 | Supported |
| H2 | Transformational Leadership → Effectiveness | 0.421 | 0.000 | Supported |
| H3 | Transformational Leadership → Satisfaction | 0.458 | 0.000 | Supported |
| H4 | Transactional Leadership → Extra Effort | 0.144 | 0.000 | Supported |
| H5 | Transactional Leadership → Effectiveness | 0.181 | 0.000 | Supported |
| H6 | Transactional Leadership → Satisfaction | 0.172 | 0.001 | Supported |
| H7 | Passive-Avoidant → Extra Effort | -0.120 | 0.005 | Supported |
| H8 | Passive-Avoidant → Effectiveness | -0.247 | 0.000 | Supported |
| H9 | Passive-Avoidant → Satisfaction | -0.141 | 0.000 | Supported |

# Supplementary Figures


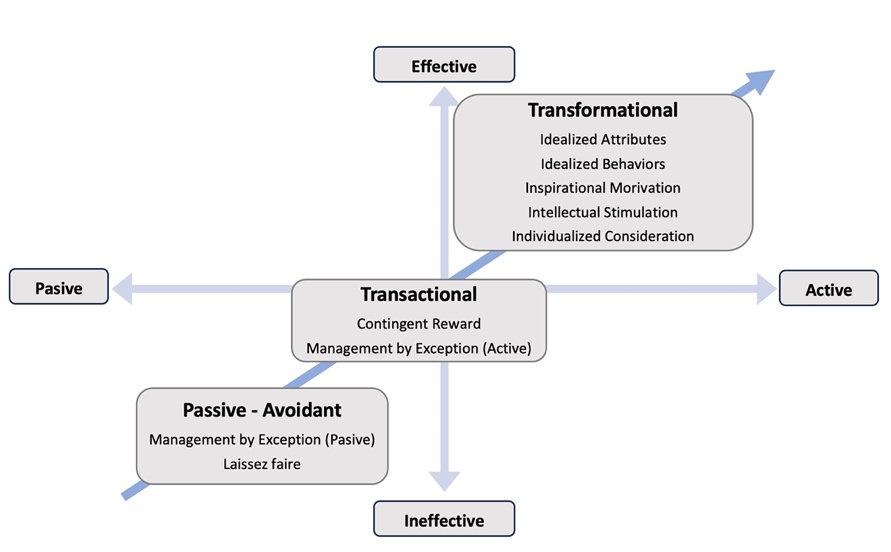


Supplementary Figure 1. Full Range Leadership Continuum (Bass & Avolio, 2011).


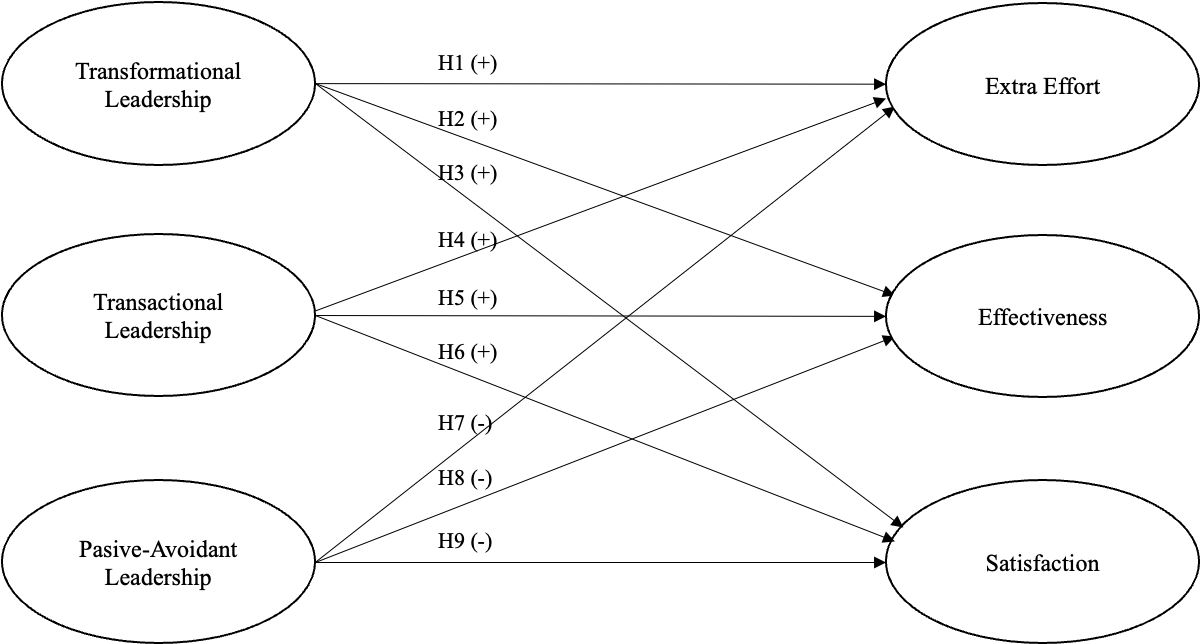


Supplementary Figure 2. Proposed Model.


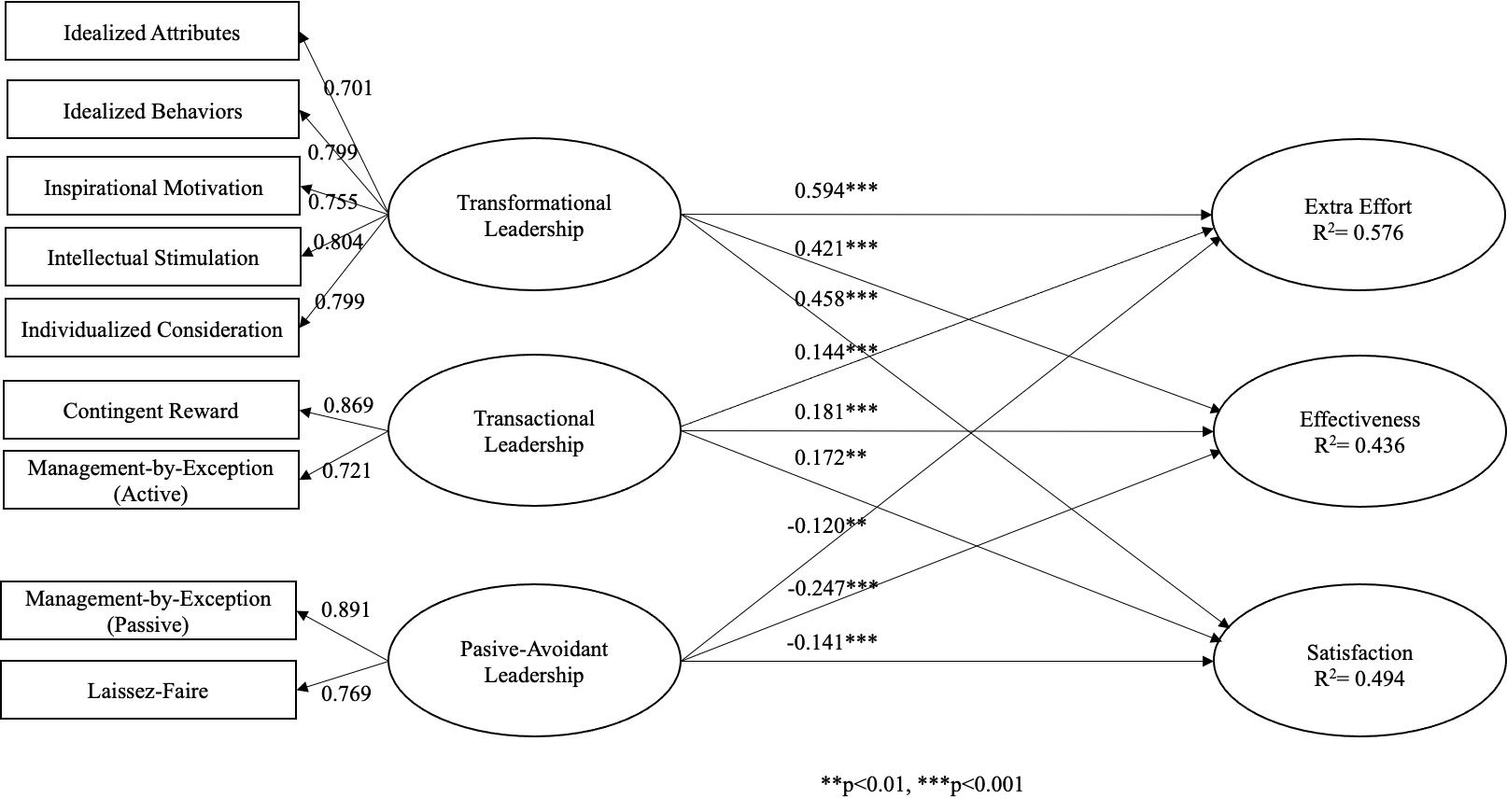


Supplementary Figure 2. Structural Model Results
